# Australian GPs’ perceptions of barriers and enablers to best practice palliative care: a qualitative study

**DOI:** 10.1186/s12904-019-0478-6

**Published:** 2019-10-31

**Authors:** Anne Herrmann, Mariko L. Carey, Alison C. Zucca, Lucy A. P. Boyd, Bernadette J. Roberts

**Affiliations:** 1 Health Behaviour Research Collaborative, School of Medicine and Public Health, Faculty of Health and Medicine, University of Newcastle, Callaghan, NSW 2308 Australia; 2 Priority Research Centre for Health Behaviour, University of Newcastle, Callaghan, NSW 2308 Australia; 3 Hunter Medical Research Institute, New Lambton Heights, NSW 2305 Australia; 4 Cancer Council New South Wales, 153 Dowling Street, Woolloomooloo, NSW 2011 Australia

**Keywords:** Palliative care, General practitioners, End of life care, Qualitative study, Implementation strategies, Barriers, Enablers, Optimal care

## Abstract

**Background:**

General Practitioners (GPs) often play an important role in caring for people at the end of life. While some international studies suggest that GPs experience a number of barriers to providing palliative care, little is known about views and experiences of GPs in Australia. This study explored Australian GPs’ perceptions of barriers and enablers to the provision of palliative care and provides new insights into how to implement best practice care at the end of life.

**Methods:**

This was a qualitative study using 25 semi-structured phone interviews conducted with GPs practising in metropolitan and non-metropolitan New South Wales, Australia. Data were analysed using qualitative content analysis.

**Results:**

GPs reported difficulties with palliative care provision due to i) the complex and often emotional nature of doctor-family-interaction; ii) a lack of evidence to guide care; and iii) the need to negotiate roles and responsibilities within the healthcare team. GPs listed a number of strategies to help deal with their workload and to improve communication processes between healthcare providers. These included appropriate scheduling of appointments, locally tailored mentoring and further education, and palliative care guidelines which more clearly outline the roles and responsibilities within multidisciplinary teams. GPs also noted the importance of online platforms to facilitate their communication with patients, their families and other healthcare providers, and to provide centralised access to locally tailored information on palliative care services. GPs suggested that non-government organisations could play an important role by raising awareness of the key role of GPs in palliative care provision and implementing an “official visitor” program, i.e. supporting volunteers to provide peer support or respite to people with palliative care needs and their families.

**Conclusions:**

This study offers new insights into strategies to overcome well documented barriers to palliative care provision in general practice and help implement optimal care at the end of life. The results suggest that researchers and policy makers should adopt a comprehensive approach to improving the provision of palliative care which tackles the array of barriers and enablers identified in this study.

## Background

### Many patients do not receive optimal palliative care

Numerous governments and non-government organisations worldwide have developed models for palliative care services to ensure efficient and effective care at the end of life [1–4]. They emphasise the importance of improving access to integrated and coordinated services that ensure that palliative care aligns with patients’ wishes [3, 5]. For example, in the UK, the “End of Life Care Strategy” was published in 2008 and has since been used to advocate for more attention to be given to end of life care [6]. National strategies to improve access to high-quality palliative care have also been used in other high-income countries, such as Australia and Singapore, and in low- to middle-income countries such as Georgia, Romania or Uganda [7]. However, international data indicates that many people with palliative care needs do not receive optimal care at the end of life, rather they experience preventable hospital admissions or receive aggressive treatments that do not increase their length or quality of life [8–10]. For example, although about 50% of all deaths occurred in a hospital [11], the majority of people would prefer to be at home at the end of their lives if possible [12–14]. It is important to understand the factors that hinder the implementation of recommended models of palliative care.

### General practitioners (GPs) may help overcome barriers to palliative care provision

There is an increasing demand for GPs to provide general palliative care services as a result of an ageing population, and limited specialist workforce capacity to meet the needs of all patients at the end of life [15].^1^ While palliative care is not necessarily their main work, most GPs provide some degree of care to palliative patients and their families [16, 17]. This includes managing side-effects of treatments and referring to secondary services that provide specialist palliative care and advice where needed [15, 18]. It has been argued that GPs have the appropriate knowledge and skillset to provide optimal care at the end of life which is often considered to be a “natural extension” of primary care [19]. Therefore, is important that we seek GPs’ views on what may hinder or facilitate the provision of optimal palliative care.

### Limited evidence on GPs’ perceptions of the barriers and enablers to providing optimal palliative care

A number of studies have explored GPs’ perceptions of barriers to the provision of palliative care. Systematic reviews have identified barriers such as time constraints [20], poor communication between healthcare providers [21] and a lack of skills [22]. Despite this, little is known about how to best overcome these barriers, and only a few studies have been conducted in the Australian context. Identified barriers to the provision of palliative care by GPs in Australia include time constraints, concerns with undertaking home visits, personal commitments, and lack of informational support from specialists [16]. Further barriers include lack of education and training in discussing end-of-life issues with patients and lack of funding for palliative care [23]. Other Australian studies have indirectly identified barriers and enablers by exploring GPs’ knowledge of palliative care [24]; and attitudes and experiences of GPs regarding palliative care provision [25–27]. The most recent Australian studies directly exploring barriers and enablers were conducted more than a decade ago [16, 23]. This indicates that more up-to-date data are required to better reflect the current palliative care landscape.

Non-government organisations (NGOs) are non-profit organisations which are independent of government influence and do not make profits to further a cultural, educational, religious or public service cause. Some NGOs, notably those focussed on cancer, have key roles to play in the development and dissemination of patient education and support services [28]. It has been suggested that NGOs may similarly support GPs’ provision of palliative care by facilitating information provision and helping patients manage their care [28]. However, the potential role of NGOs has not been well explored in relation to palliative care. Further exploring GPs’ views about what may hinder and assist with palliative care provision can help ensure that best practice care is being incorporated into their routine practice.

### Aims

To explore Australian GPs’ perceptions of barriers and enablers to the provision of palliative care.

## Methods

### Design

This was a qualitative descriptive study [29]. Twenty-five semi-structured phone interviews were conducted with GPs practising in New South Wales (NSW), Australia.

### Study context

In Australia, patients are able to claim a Medicare rebate for range of services, provided by GPs and practice nurses. Medicare is a national, tax-funded scheme which is administered within a national framework and gives Australian residents access to healthcare [30]. The Medicare Benefits Schedule (MBS) lists all the Medicare services subsidised by the Australian Government. The Pharmaceutical Benefits Scheme (PBS) includes all the medicines available to be dispensed to patients at a government-subsidised price [31]. Medicare rebates partly cover the cost of medical services and are commonly paid in the form of a percentage of the benefits schedule fee [31]. GPs are often the first point of contact for people who feel sick or have a health concern and treat a wide range of medical conditions and health issues [32]. In NSW from 2017 to 2018, there were 11,169 practicing GPs [33].

### Recruitment

We used stratified sampling which means that we divided all eligible participants into different subgroups and then randomly selected participants proportionally from the different subgroups. GPs currently practising (either full- or part-time) in community general practices in NSW, Australia were eligible. GPs who were no longer practising (e.g. retired), or not practising in New South Wales were ineligible (e.g. changed practice address). GPs were identified through the Australasian Medical Publishing Company (AMPCo) database. A letter of invitation containing a study information sheet, indication of interest form and reply paid envelope was sent to a stratified random sample of 240 practitioners. All eligible GPs were divided into two subgroups: those with practice addresses classified as non-metropolitan and those with practice address classified as metropolitan according to the Accessibility/Remoteness Index of Australia [34]. Within each subgroup 120 GPs were selected randomly. This ensured that the different subgroups of GPs were adequately represented within the study sample. All consenting GPs were recruited to this study. A follow-up telephone call was made to non-responders after 2 weeks. Most GPs did not provide a reason for not consenting to the research invitation. However, 12 GPs who provided a reason indicated they were either not interested (*n* = 6); too busy (*n* = 5) or they had no palliative care patients (*n* = 1). Study participants provided informed verbal consent prior to commencing the interview. Recruitment and data collection were conducted between November 2017 and April 2018.

### Data collection

Interviews were conducted by two researchers (AH and AZ), recorded and transcribed. The interview guide involved questions which were asked in a set order, with probes used as required to elicit details on topic areas not initially spoken about by the participants. The questions and the probes were informed by a literature review and discussions amongst the research team. An expert panel of four health behavioural researchers, a cancer policy analyst and a cancer supportive care service manager reviewed the interview questions. At the end of the interview, participants were asked questions about a range of sociodemographic characteristics (see interview guide in Additional file 1). Standardised questions were used wherever possible. Recruitment was carried out until further data gathering was not perceived to provide any additional information to help answer the research question.

### Data analysis

An inductive content analysis approach was used to analyse the data and ensure all relevant codes were captured [29, 35]. Each transcript was coded by one researcher and all codes were double-checked by another member of the research team. Initial coding was conducted by MC, AZ, LB and AH. Based on the initial codes, more abstract categories were developed and a coding matrix was derived which was reviewed by all members of the research team. Thus, the first interviews were used to form the coding matrix. Codes and categories of the later interviews were assigned to this coding matrix. If a code or category did not fit into the matrix, a separate code or category was developed to ensure all data was captured, regardless of whether it fitted into the existing model. This helped us to validate and extend conceptually the coding matrix. Codes and categories were refined until agreement was achieved. Threads of meaning (i.e. themes) were developed across categories to allow for interpretation of manifest and latent content and help structure the presentation of the study results. Demographics are presented using appropriate summary statistics.

### Ethical approval

Ethical approval was sought and approval received on 9th October 2017 from the University of Newcastle Human Research Ethics Committee (approval number: H-2017-0304). GPs were informed that participation in the research was voluntary and that their data would be kept confidential. Interviews were conducted in auditory privacy. The audio files were saved in password protected folders. Transcripts were de-identified immediately upon receipt.

## Results

### Sample characteristics

Of the 240 GPs invited to participate, 229 were eligible. A total of 11 GPs were ineligible for various reasons, including no longer practising at their recorded practice and a new practice was not known (*n* = 9) and no longer practising as a GP (*n* = 2). 13 GPs consented after the initial invitation and a further 12 after receiving a reminder follow up call. This gave a total of 25 GPs who completed a telephone interview (response rate: 11%). The interviews lasted between 23 and 60 min. As shown in Table 1, just over half of the participants were male, and 63% were aged 50 years or over. Non-metropolitan GPs were more likely to participate (28% metropolitan versus 72% non-metropolitan; χ^2^ (3) = 5.4; *p* = 0.02). We perceived that a point of saturation occurred after conducting 15 interviews. The following interviews helped to confirm the themes.

**Table 1:** Participant characteristics

	Mean (range)	*n*	(%)
Gender (*N* = 25)			
Male		13	(52)
Female		12	(48)
Age (years) (*N* = 25)	51 (34–62)		
30–39		3	(12)
40–49		7	(28)
50–59		11	(44)
60–69		4	(16)
Practice location (*n* = 25)			
Major city		7	(28)
Inner Regional		13	(52)
Outer Regional		4	(16)
Remote		1	(4)
Years practising as GP (*n* = 25)	19 (3–38)		
< 10		7	(29)
10–19		4	(17)
20–29		5	(21)
30 or more		8	(33)
Hours worked per week (*n* = 25)	39 (21–60)		
< 35		9	(36)
35–44		7	(28)
45–54		6	(24)
55 or more		3	(12)
Travel time to work one way (minutes) (*n* = 25)	16 (3–75)		
< 10		9	(36)
10–29		12	(48)
30–59		3	(12)
60 or more		1	(4)
Employment (*n* = 25)			
Principal		10	(40)
Employee		5	(20)
Associate or Contractor		10	(40)
Language spoken when practising (*n* = 25)			
English only		21	(84)
English and other		4	(16)
Post-graduate education in palliative care (*n* = 25)			
Yes		1	(4)
No		24	(96)
Country of medical degree (*n* = 25)			
Australia		19	(76)
Other		6	(24)
^#^GP Fellowships (*n* = 25)			
Royal Australian College of GPs (RACGP)		19	(76)
Australian College of Rural and Remote		2	(8)
Medicine (ACRAM)			
No		6	(24)

^#^Totals add to more than 100% as GPs may be fellows of more than one college (i.e. associations that bring together medical practitioners of a particular medical subspecialty or geographical area)

### Findings

Analysis of the data identified seven themes: GPs in the present study reported struggling with the complexity of palliative care and system-related barriers to optimal care provision. They identified a number of strategies to overcome these challenges, such as adequately managing time pressure, facilitating multidisciplinary teamwork, fostering the uptake of guidelines, further education on palliative care, and using non-government organisations (NGOs). These themes are described in detail below.

### Complexity of palliative care provision as challenge

Many GPs in this study perceived palliative care to be time consuming due to a number of reasons, including i) the complex and often emotional nature of doctor-family-interaction, and ii) a lack of evidence to guide care; and iii) the need to negotiate roles and responsibilities within the healthcare team. These reasons are discussed in detail below.

Some GPs experienced palliative care as emotionally draining and time consuming. They reported a degree of emotional attachment to the patient and their family. Many felt that “switching back” from seeing a person with palliative care needs to the daily routine of primary care can be difficult, particularly when palliating younger people and dealing with their families’ grief.

*“There is a lot of the emotional involvement with it [=caring for the patient and family] and note taking and making sure that you're doing everything right.” (male, 50 years)*

These emotional components added to GPs’ perceived time commitment. GPs felt that high quality palliative care requires lengthy conversations with patients and their families to elicit their preferences and tailor care accordingly. Logistic issues with scheduling family meetings or phone conferences to discuss the recommended care may also add to the challenges of providing palliative care.

*“Well, I think sometimes it can get very complex. Especially these days. I mean, not only do you have partners, but sometimes you have new partners, old partners as well. Then you have all the children and the step children. [ … ] They all care about what's going on.” (female, 55 years)*

Particularly difficult discussions were perceived to be about when to initiate end-of-life care or how to discuss treatment options with patients who deny their palliative condition. According to the GPs in this study, such discussions were often compounded by a lack of evidence for many palliative care treatments. GPs felt that they were often delivering care “outside of the evidence base” which resulted in an abundance of decisions that involved no single “right” treatment approach from a medical point of view. This means that delivering palliative care often involves GPs finding solutions for problems as they arise, rather than adhering to pre-existing protocols.

*“That's especially around that when is it palliative and when is it not, as you make that transition which is a grey zone. How do you manage the de-prescribing and the cessation of what were previously thought to be therapeutic treatment regimens? Because you're nearly always outside of the evidence base.” (male, 56 years)*

GPs also reported that palliative care involves a number of healthcare services, including nurses, palliative care specialists, or, when working in an Aboriginal Medical Service, Aboriginal Health Workers. GPs indicated that there is often a degree of uncertainty with regard to who is responsible to provide which service to patients and their families. They felt that negotiating and co-ordinating between services often became part of their role, adding to the complexity and time required to provide palliative care. However, some GPs explained that although time commitments were a challenge, this was often due to their attitudes to care provision, rather than a lack of resources. Many perceived it as their duty of care to invest sufficient time to ensure optimal care at the end of life. These GPs indicated that it was important to remember that patients’ needs should override doctor-related barriers, such as being time poor or practising far away from the patient’s home.

*“I sometimes inherit patients from other doctors, they are too lazy to do home visits. It really annoys me when that happens. [ … ] I think, well is time a factor? Well I suppose if you’re really busy, time is always a factor no matter what you do. But I think that when people are dying … what can you say? Well I’m not going to say, well I’m too busy, I can’t come today.” (male, 62 years)*

### Managing time pressure to ensure the provision of optimal palliative care

GPs listed a number of strategies to help deal with their workload. For example, they indicated that effective and flexible practice management could help restructure a GP’s busy schedule to facilitate palliative care provision. They noted that longer, fixed and reoccurring appointments, using telephone and online communication instead of face-to-face contact, and offering emergency clinics could help incorporate palliative care into GPs’ daily routine. Specifically, GPs named mechanisms to help incorporate home visits into their clinical practice, including scheduling home visits after the clinic appointments so that they could conduct these visits on their way home. One GP also suggested that the provision of home visits to existing patients who live within a defined geographic area should be a requirement for GP accreditation. A number of GPs further recommended involving nurses in the provision of home visits to distribute workload.

*“When I would go on one visit I would book to go and see them in two or three days so gave a bit of notice. Then I'd go back to the office and I'd block out to a chunk of time. [ … ] Then, I suppose how it fitted in mostly is either at the end of the day or at the beginning of the day, that is if something came up and they needed either to see me that day or the next.” (female, 60 years)*

Many GPs indicated that increasing their experience with providing palliative care could shorten care provision time, for example, by increasing GPs’ skills in symptom management and eliciting patients’ preferences. A number of GPs stressed the importance of these strategies and highlighted the benefits of GPs making themselves available for their patients, rather than putting boundaries around their availabilities. However, few GPs also noted that there may be a need to set some boundaries, for instance by providing patients with the telephone number of another healthcare provider in case the treating GP cannot pick-up the phone.

*“If I've got someone dying of cancer, I usually give them my mobile phone number and I say if I don’t answer it, you'll know to ring whoever's on call, but in general, I'd answer it. It's amazing how rarely the patients ring me after hours. Because knowing that I'm there if they need me. I would do a house call at 2:00am if they want me to. It does them a lot of good.” (male, 58 years)*

### System-related barriers

GPs felt that it was important to elicit, and flexibly respond to, the changing needs of patients and their families but that current models of care sometimes promoted eclectic, process-centred, rather than patient-centred care. A number of GPs noted that healthcare services are funded by the government to provide care for patients within a particular geographic region. Thus, there are rules about how far government-funded palliative care service providers can travel and some patients living in remote areas may miss out on timely care provision. Some GPs indicated difficulties with obtaining admitting rights to a hospital. They reported that it may take them a couple of years to undergo all required accreditation procedures to be able to admit patients to a hospital. Also, a number of GPs perceived a lack of reward for their time commitment. They felt that government rebates were insufficient to cover the care provided. For example, longer consultations which were required for many patients were not considered profitable given the rebates available for longer consultations. GPs suggested to include palliative care as separate item on the MBS to more realistically reflect the amount of time that providing palliative care commonly takes.

*“I think the way general practice has been paid over the last few years has resulted in a general decline in the quality of care. [ … ] I think because of the way the MBS works and because of the freezing of Medicare [rebates] and because of the poor general remuneration in general practice, the way to make a living in general practice is to see lots of people for short periods of time.” (male, 59 years)*

Affordability of care was also discussed from the patient perspective: Many GPs reported that they tend to bulk bill people with palliative care needs.^2^ However, some medications they would like to prescribe are only approved under the PBS for particular conditions so may not be rebated. For example, ondansetron can be prescribed as an antiemetic for people with cancer, but not for other conditions where nausea and vomiting may be a persistent problem. Thus, GPs recommended to change the scheme to allow doctors to prescribe these drugs for palliative purposes. A number of GPs also expressed dissatisfaction with the complexity and uncertainty of funding for palliative care resources. They reported a lack of clarity about which government services are responsible for funding a patient’s community care, and that this uncertainty can add to their workload as multiple phone calls may be required to determine care providers and costs. Some GPs suggested that one solution may be for palliative care to have its own budget and not be split between jurisdictions.

*“I hate bureaucracies, but somehow putting palliative care to have its own bucket of money so that it can work out what to do in the current climate might be the only way of doing it.” (female, 56 years)*

### Challenges to multidisciplinary teamwork

GPs indicated that palliative care requires the collaboration of various healthcare providers. However, many GPs indicated that this collaboration is often characterised by “token” multidisciplinary teams who only co-exist rather than efficiently and effectively work with each other. This was for a number of reasons: First, GPs felt that there is often an “us and them” mentality between GPs and palliative care specialists due to perceived differences in knowledge and skills. Some GPs reported that specialists appeared to doubt GPs’ ability to serve as central palliative care providers.

*“But it's the systems and processes that are not working at the moment. I think that they need to include general practice. I think it's that mistrust. There is a basic mistrust of GPs in our area, I feel. They [=palliative care teams] think we don't know much and that they can't trust us to make decisions about some of this stuff.” (female, 53 years)*

Many GPs also noted that patients may perceive a value in specialist care with the term “specialist” making them feel as though they are getting better care than that provided solely by a GP. Thus, some GPs felt under pressure to involve specialists in palliative care even if they felt competent to provide the required care.

GPs also noted an unequal distribution of workload, given that not all GPs may be willing or confident to provide palliative care. For example, they may lack skills in clinical assessment and communication. Consequently, people with palliative care needs may be “offloaded” to GPs who are willing to take on new clients when they are at the palliative stage. A number of GPs felt that existing palliative care protocols were an additional barrier as they were not perceived to include GPs and focus on “streamlining” (female, 53 years) palliative care, rather than involving the myriad of healthcare providers required to address the range and complexity of patients’ needs. GPs also noted that communication between healthcare providers often lacks documentation of important details, for example when patients transition from hospital to home and discharge summaries are not adequately communicated to the treating GP.

*“Now, in region X all use different computer systems even inside hospitals. They can't even talk one hospital to another electronically, let alone communicating in the community. [ … ] They [=palliative care teams] are going in visiting, changing a syringe driver or something, maybe doing a pain score. The patient tells me they’re having a more extended conversation, but there’s nothing to document that. They go back and write in their notes at palliative care. I visit and write in my notes on the computer, because we’re computer-based, and the communication isn’t ideal.” (female, 56 years)*

Thus, GPs often felt left out and unappreciated. Poor communication may also have downstream effects on family members and can add to GPs’ time burden as they may have to follow-up with other healthcare providers and help ensure that patients and their families have an accurate understanding of the patient’s care.

### Strategies to facilitate multidisciplinary teamwork

GPs indicated that in order to ensure efficient and effective multidisciplinary teamwork there is a need to improve communication processes and build relationships between various healthcare providers. They recommended communication to be detailed, for example by having comprehensive notes in electronic format that are compatible with GPs’ medical records. Some GPs suggested developing an online platform to support communication between medical staff and patients, and between patients and supportive others who may be living far away. This could help increase communication from being simple reporting to mutual exchange and discussion. It may also help provide a clear definition of roles and responsibilities within the team to ensure healthcare providers, patients and their families have a good understanding of the relevant points of contact.

*“They [=multidisciplinary teams] are having a [n online] platform where they can communicate to families. There is a support network and afterhours service for the patients, but also a clear contact from the GP point of view to the palliative care team. Yeah, I think that’s pretty much it, I reckon.” (male, 34 years)*

Many GPs suggested a model of care that involves GPs as central care coordinators who consult other healthcare providers, such as nurses or specialists, if required. GPs felt that each healthcare provider has variations in the way they practice which can confuse patients and may lead to inefficient care. GPs perceived themselves to have the right knowledge and skillset needed to co-ordinate palliative care, act as the conduit between patients, families and healthcare providers and help ensure continuity of care. GPs also suggested that the provision of day-to-day care could be supported by palliative care nurses who could play an important role in reporting issues that need to be addressed by the GP.

*“Well, you know, probably the key resource for me is a palliative care nurse. They're just bloody invaluable.” (male, 42 years)*

### Fostering the uptake of guidelines and further education on palliative care

Most GPs used general medical rather than palliative care specific guidelines and some reported that they lacked knowledge about available palliative care guidelines and toolkits. GPs felt that palliative care skills were gained by doing and that palliative care followed similar pathways to primary care.

*“The guidelines that I frequently refer to would be things like opioid conversion charts. [ … ] Most people do run into the same problem and it's just like a GP will have a handful of drugs that they will use commonly in high blood pressure and heart failure. In palliative care you tend to find the same sort of pathways you use as far as what you try in what order.” (male, 58 years)*

Some GPs were interested in further education on palliative care, while others felt that they had sufficient experience and/or training. Many GPs reported that they often do not have the time to attend courses during their work time, or that they may not always be aware of available opportunities for education on palliative care. They also noted the lack of standardisation and accreditation of palliative care education as barrier to further education. GPs suggested some strategies to increase palliative care education for primary care. This included integrating palliative care training into routine clinical practice and providing engaging and interactional “hands on learning” and mentoring, rather than formalised education based on textbooks.

*“I suppose having a mentoring, like with an experienced palliative care provider being able to link in individually with their GP who could just be there if you had questions, I thought that might be helpful.” (female, 53 years)*

This could occur with the help of small group problem-based learning and having an experienced GP or palliative care specialist visit the practice to discuss their clinical experience and positive aspects of palliative care provision. Such training would involve local palliative services to help GPs network and get a better sense of locally available services. Some GPs said that this could be delivered as compulsory in-house professional education.

*“There are early morning palliative care meetings. I think once a month or so when the specialist comes up. He also does like a training session. [ … ] It's usually about seven in the morning and we have breakfast with them. But they are very good at giving information and if you're new to the area it's very good to be able to go along to these meetings.” (female, 55 years)*

Many GPs reported that education programs should involve repeated information sessions, during and out of business hours, with additional follow-ups to allow for further discussion. Online education may be particularly relevant for GPs working in rural settings given potentially considerable travel times. Some GPs suggested promoting palliative care as a GP sub-speciality and promoting palliative care training for GPs, clarifying the roles and responsibilities of GPs and facilitating multidisciplinary teamwork. A number of GPs felt that these strategies could help ensure that GPs keep up to date with the latest clinical practice guidelines and increase their confidence in delivering palliative care, which may ultimately be an incentive to take on more patients who require palliative care.

*“There are GPs who will do lots of skin things and they can go and do courses. Other ones who provide women's health and children's health. There are all these courses that you can go and do before you want to do, it's like a sub specialty. It's not quite that clear in palliative care yet.” (female, 53 years)*

### Non-government organisations (NGOs) as enabler for optimal palliative care

GPs indicated that NGOs could provide important support with delivering palliative care to patients and their families. For example, NGOs could help facilitate patient and carer support. GPs discussed the concept of having an “official visitor”, i.e. someone who provides peer support or respite, for example by sitting with the patient, so the carer can do their daily tasks, such as grocery shopping. Trained volunteers may also be able to help with logistic issues, for example by providing transport services. They suggested that NGOs could facilitate the training of such “official visitors”.

*“More emotional distraction I suppose. In hospitals people who used to go in and change the water of the flowers each day. They'd be retired, usually older women who would just go in and have a bit of a natter with, a talk with the patient and change their water. I didn't know whether you could have like a visitor who'd go around to some of the people in the area and just have a cup of tea with them. Just break up their day and give them someone else to talk to who cared about them but wasn't in a caring role, wasn't going to ask about, apart from how you feeling today, wasn't really going to delve any more” (female, 53 years)*

*“Volunteer training. Expanding volunteers to provide assistance and support to people that are dying at home. I think that's a great role for NGOs.” (male, 58 years)*

Some GPs also reported that NGOs could help communicate existing services to patients and families by providing them with locally tailored, take-home information, to back up face-to-face consultations with their healthcare providers. This would involve trustworthy written information that people can read at their convenience to help them understand the information covered in the consultation, for example related to the timing of transitioning from curative to palliative care.

*“Maybe somebody who can spend more time talking through that [=medications], what it might actually look like, what it might be like, when you would give the medication, and how you would judge the response to that, would be a worthwhile investment, potentially if you had [it] in the same way that you have cardiac and pulmonary rehab nurses. Actually if you’re from that service, you’d prevent acute admissions and therefore it’s a cost-effective service as well as being good for holistic care.” (female, 56 years)*

GPs explained that carer support could also involve bereavement counselling. This is often offered at hospices but travelling to and from the hospice may pose a significant time burden on some people. Thus, a service that *“could offer counselling in the home may be helpful” (female, 56 years)*. Some GPs thought that NGOs could further support patients with navigating the healthcare system. They elaborated on the breast care nurse model which could be adapted for palliative care. This may involve a person that patients and families can talk to in order to help them access the right care at the right time. It may also involve telephone support on physical symptom management, such as help with medications that are not working and how to deal with symptoms.

*“So if someone diagnoses palliative care person X, there could then be the palliative care nurses modelled around a similar person. I know people who have breast cancer and that system works extremely well. So I think rather than try and reinvent the wheel, you could extrapolate that sort of system.” (female, 36 years)*

GPs felt that NGOs could further support healthcare providers by ensuring access to palliative care specific clinical pathways which could be a *“cross between clinical guidelines and referral pathways” (male, 59 years)*, and could be delivered via smartphone apps to ensure ease of use. This may include the provision of central points of contact, such as phone numbers of palliative care services in a specific geographic area. GPs felt that NGOs could also help increase communication between healthcare providers, for instance by facilitating multidisciplinary team meetings through online communication platforms.

*“It's always handy to have a central resource that can direct you to all the various things that are appropriate. Maybe the Cancer Council would be a good central resource group.* 3 *It's often the first stop we go to for information about cancer. So it would be understandable if they took a specific interest in palliative care. [ … ] On the website where you can click on a tab that says palliative care, and then under that's a tab saying ‘resources’, ‘palliative nurse agencies available’, and various things that are relevant. Local areas, et cetera, et cetera.” (male, 59 years)*

Simplified guidance on advance directives to be used in the primary care setting was seen as further enabler. GPs felt that there are lots of advance care planning tools available but that the level of information provided by these tools is often overwhelming which may be a barrier to some patients using them. GPs indicated that NGOs could work with healthcare districts to provide simplified tools and prompts to facilitate discussions on advance directives, as well as locally tailored training to promote awareness and use of such tools among GPs.

*“Then the New South Wales advanced care directive document that they have is quite a lengthy one, but I’ve had that sent back to me by a patient this morning, who isn’t particularly elderly or unwell, but definitely wanted to have an advanced care directive. She downloaded a different one, because she said that the New South Wales one was too lengthy. Ten pages with too many different detailed questions.” (female, 46 years)*

GPs reported that NGOs could foster campaigning to promote palliative care to GPs. They felt that palliative care is often “invisible work”, and that promoting palliative care at conferences or other events may help increase GPs’ interest and willingness to engage in this branch of medicine. GPs also indicated that it would be important to educate the public on GPs’ key role in palliative care which is currently still often undervalued and under recognized. GPs felt that it was important to change community perceptions to ensure that they understand that patients are not an inconvenience to GPs and their families if they prefer to be visited or die at home.

## Discussion

The present study reports on GPs’ perceptions of barriers and enablers to optimal palliative care provision. While there has been much research on barriers, there has been less focus on enablers, and in particular, the potential role of NGOs in improving palliative care service provision. The current study offers new insights into the potential solutions to overcoming well documented barriers to palliative care provision and help implement optimal care at the end of life.

### Overcoming personal barriers to palliative care provision

GPs in the present study explained a number of barriers which have been reported previously, such as lack of time [16, 36, 37], lack of clarity of roles and responsibilities of GPs and other healthcare providers and services [38], poor communication between healthcare providers [16, 37, 39, 40] and poor remuneration [16, 41, 42]. In line with previous studies [43, 44], the findings also suggest that not all GPs are aware of the resources and guidelines available to support optimal, evidence-based palliative care provision. Similarly, GPs may not be aware of all drugs that could be used for the provision of palliative care and that are listed in the Pharmaceutical Benefits Scheme. Further, some GPs reported concerns about the lack of access to ondansetron via the PBS for people with conditions other than cancer. These concerns may reflect a lack of awareness of similar medications available to palliative patients, such as metoclopramide. Consistent with previous research, these findings suggest that GP knowledge may also be a barrier to the provision of optimal palliative care [16, 41]. More efforts should be made to improve the dissemination and implementation of the evidence and guidelines available to support palliative care provision.

In addition to these previously reported barriers, personal barriers, such as the emotionally draining nature of palliative care work, were reported by some GPs in this study. This highlights the need to ensure that GPs, who may not operate within a team environment, have access to the types of supports, such as debriefing, which are more commonly available for healthcare providers working in teams [45]. Additionally, GPs reported interpersonal difficulties with other providers involved in the care of palliative patients, including a lack of understanding and acknowledgement of the GP’s role. This suggests that development and communication of palliative care pathways, including discussion of how these may be implemented in local contexts is important to supporting collaborative relationships between professionals. For example, it may be helpful if palliative care guidelines more clearly outline the roles and responsibilities of each healthcare provider within multidisciplinary teams caring for people with palliative care needs. Such improved guidelines may also include further decision support for how to find the “right” palliative care for each patient as GPs in the present study felt that they were often delivering care “outside of the evidence base” which resulted in an abundance of difficult preference-sensitive decisions.

It is notable that while the GPs in this study had suggestions for improving the use and accessibility of guidelines, many felt they had the necessary medical knowledge to provide palliative care. This finding may reflect sampling bias, for example, the GPs in this study may be more interested in and active in palliative care provision than those in other studies. Or it may indicate that while specific knowledge gaps reported elsewhere may exist [16, 41], these were not perceived to be a major barrier to palliative care provision. This aligns with previous findings suggesting that GPs perceive palliative care as a “natural extension” to primary care and generalist, rather than specialist care, which may only require occasional specialist advice that is readily available when needed [46, 47].

### Need for comprehensive policy approach

Palliative care is complex and involves interactions between GPs and patients and their families, but also among GPs and other service providers both in the community and acute setting [48]. For GPs, it is also delivered within the context of providing care for patients with a wide range of conditions and needs. Therefore, it is perhaps unsurprising that the GPs in the present study identified various strategies needed to support palliative care provision. These spanned from policy solutions, such as increased remuneration and ensuring that palliative care workload was shared by making it a requirement for accreditation; to workforce solutions, such as improving the accessibility of palliative care nurses and the use of technological solutions to aid in communication. The array of enablers identified in this study suggests that policy makers at a national level need to pay more attention to these topics and implement a comprehensive approach to improving the provision of palliative care which tackles difficult and costly issues, such as remuneration and workforce capacity. This contrasts with much of the previous intervention work aimed to improve palliative care provision which focusses heavily on single issues, such as GP knowledge [49, 50].

### Strategies to facilitate multidisciplinary teamwork

Some GPs in this study suggested that relationships between members of the healthcare team could be strengthened through further education on palliative care. This could be delivered through mentoring and locally tailored “hands on” learning, rather than formalised education based on textbooks. In line with previous studies, GPs recommended to have an experienced GP or palliative care specialist visit the practice as part of in-house training and discuss their clinical experience and positive aspects of palliative care provision [51, 52]. Such training may allow GPs to better understand locally available services and how to access them.

Further education may also be targeted at practice nurses as GPs indicated that effective and flexible practice management for example by scheduling reoccurring, sufficiently long appointments could help facilitate care provision. They suggested to provide palliative care within fixed and reoccurring appointments and using telephone and online communication instead of face-to-face contact. This is in line with previous efforts to improve palliative care provision. For example, in Australia, telephone conferencing between GPs and specialists has been funded by the MBS and has been shown to be a time-effective communication tool [53]. Some GPs felt that online platforms could be specifically designed to support communication between various healthcare providers, patients, and their families who may be living far away. Research into chronic illness populations suggests that such technology may facilitate high-quality and affordable healthcare by storing all relevant medical information in one central place. This would allow transparent communication and collaboration across all members of the healthcare team, even if they are locally distant, and actively engaging patients in their healthcare [54, 55]. Further research is needed to develop and test online platforms specific to the context of palliative care provision.

### NGOs as palliative care advocates and promoters of “official visitors”

GPs in this study recommended various roles for NGOs, including educational initiatives focussed on raising the profile of palliative care among GPs as many of the participants felt that palliative care is “invisible work”. GPs also recommended providing simplified guides on palliative care pathways and assistance with advance care planning to which GPs can refer. This is in line with previous studies suggesting that advance care planning tools may be too complex for patients and GPs to easily integrate into patient care [56].

Most of the recommendations GPs in this study made focussed on ways that NGOs could support patients and carers via volunteer-delivered peer support and respite initiatives, as well as assistance with service navigation. These types of services mirror those which are commonly delivered by cancer organisations. In particular, Cancer Council Australia has a suite of volunteer peer support programs which link cancer patients with a peer who has completed cancer treatment. In the context of palliative care, a peer who has had experience supporting a loved one through palliative care could be trained to provide support to current patients and carers. These peers could be volunteers acting as “official visitors” who visit the patients at home, provide distraction and psychosocial support, and give the carer some time to do daily tasks, such as grocery shopping or household work. The data suggest that some GPs support an adaption and better dissemination of existing services to better cater for the needs of patients who are receiving palliative care.

### Limitations

GPs practising in metropolitan and non-metropolitan areas were recruited to include the perspectives of those across a variety of geographical locations. However, the study involved participants from only one Australian state and GPs in other states may have different perceptions of the barriers to optimal care provision. They may also have different suggestions for how to improve palliative care. Interviews were conducted by telephone rather than face-to-face. While it is possible that face-to-face interviews may have resulted in richer data [57, 58], telephone interviews were considered the most feasible method for this study. It reduced travel burden for research staff and enabled greater flexibility in the timing of interviews. This was considered important given GPs’ high workload and time pressure. While the response rate was low (11%), it is similar to other studies which have used comparable methodology [59–61]. Finally, GPs who participated in this study may be more likely to engage in palliative care provision. Thus, consent bias may have occurred.

## Conclusions

This study offers novel insights into GP’ perceptions of barriers to palliative care provision. It is also one of few studies to describe strategies to help implement best practice care at the end of life. The results suggest that a comprehensive approach to improving the provision of palliative care is required which tackles the array of barriers and enablers identified in this study. Specifically, GPs in the present study suggested to increase relationship building between various healthcare providers involved in palliative care provision and to improve skills and confidence in palliative care provision through mentoring and locally tailored further education. GPs also recommended that palliative care guidelines more clearly outline the roles and responsibilities within multidisciplinary teams caring for people with palliative care needs. They indicated that online platforms could facilitate communication between healthcare providers, patients and their families and provide centralised access to locally tailored information on palliative care services and how to access them. This study is also one of the first to explore how NGOs could assist GPs in improving palliative care, e.g. by raising awareness of the key role GPs can play in palliative care provision and implementing the concept of having an “official visitor”, i.e. a volunteer who provides peer support or respite to patients and their families.

## Data Availability

The datasets used and/or analysed during the current study are available from the corresponding author on reasonable request.

## Abbreviations

AMPCo: Australasian Medical Publishing Company
GP: General practitioner
MBS: Medicare Benefits Schedule
NGO: Non-government organisation
NSW: New South Wales
PBS: Pharmaceutical Benefits Scheme

## Footnotes

1 A GP is a medical doctor who is qualified in general medical practice. GPs are often the first point of contact for someone, of any age, who feels sick or has a health concern. They treat a wide range of medical conditions and health issues.

2 Bulk billing involves healthcare service provision without any out-of-pocket expenses for the patient, i.e. the provider bills the Australian government-funded Medicare scheme directly and accepts the Medicare rebate as full payment for their service.

3 The Cancer Council is an Australian charity organisation undertaking and funding cancer research and other activities aiming to prevent cancer, provide information and support for people affected by cancer, and advocate for changes to reduce cancer risk and improve access to care and treatment.
